# Supplementary material for: Carbon p Electron Ferromagnetism in Silicon Carbide
Source: Sci Rep. 2015 Mar 11;5:8999. doi: 10.1038/srep08999 (PMC4355737; doi:10.1038/srep08999)
Supplement: Supplementary Information [file srep08999-s1.pdf]

Carbon *p* Electron Ferromagnetism in Silicon Carbide

Yutian Wang[1,5], Yu Liu[1,2], Gang Wang[2], Wolfgang Anwand[3], Catherine A. Jenkins[4], Elke Arenholz[4], Frans Munnik[1], Ovidiu D. Gordan[6], Georgeta Salvan[6], Dietrich R. T. Zahn[6], Xiaolong Chen[2], Sibylle Gemming[1,6], Manfred Helm[1,5], Shengqiang Zhou[1]

1. Institute of Ion Beam Physics and Materials Research, Helmholtz-Zentrum Dresden-Rossendorf, Bautzner Landstr. 400, 01328 Dresden, Germany
2. Research & Development Center for Functional Crystals, Beijing National Laboratory for Condensed Matter Physics, Institute of Physics, Chinese Academy of Sciences, Beijing 100190, China
3. Institute of Radiation Physics, Helmholtz-Zentrum Dresden-Rossendorf, Bautzner Landstr. 400, 01328 Dresden, Germany
4. Advanced Light Source, Lawrence Berkeley National Laboratory, Berkeley, California 94720, USA
5. Technische Universität Dresden, 01062 Dresden, Germany
6. Institute of Physics, Technische Universität Chemnitz , 09107 Chemnitz, Germany

Supplementary materials

Section 1: Transition metal free SiC substrates

To exclude the presence of possible transition metals (Fe, Co and Ni) in SiC substrates, we have performed Particle-induced X-ray emission (PIXE) using 2 MeV protons with a broad beam of 1 mm^2. The result is shown in Fig. S1. As stated in ref.

1, PIXE is a sensitive method to detect trace impurities in bulk volume without structural destruction. In the spectrum, the narrow peak is from Si K-line X-ray emission. The broad peak is due to the Secondary Electron Bremsstrahlung background. If there are any transition metal impurities, they are below the detection limit of around 1 µg/g.

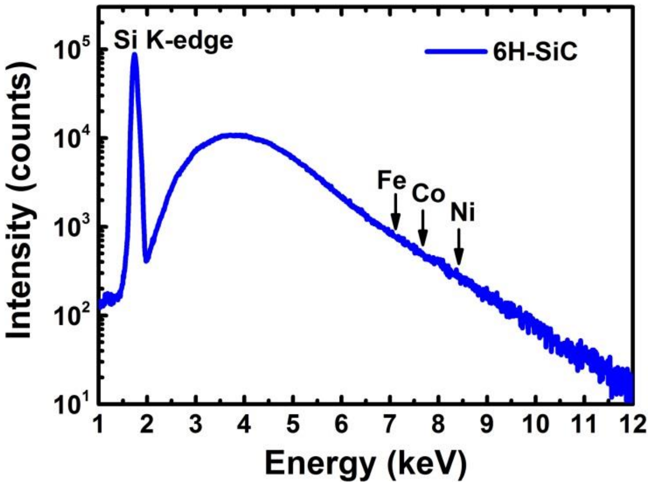

Figure S1: PIXE spectrum for the 6H-SiC wafer by a broad proton beam. Within the detection limit, no Fe, Co or Ni contamination is observed.

Section 2: Magnetic properties of pristine SiC

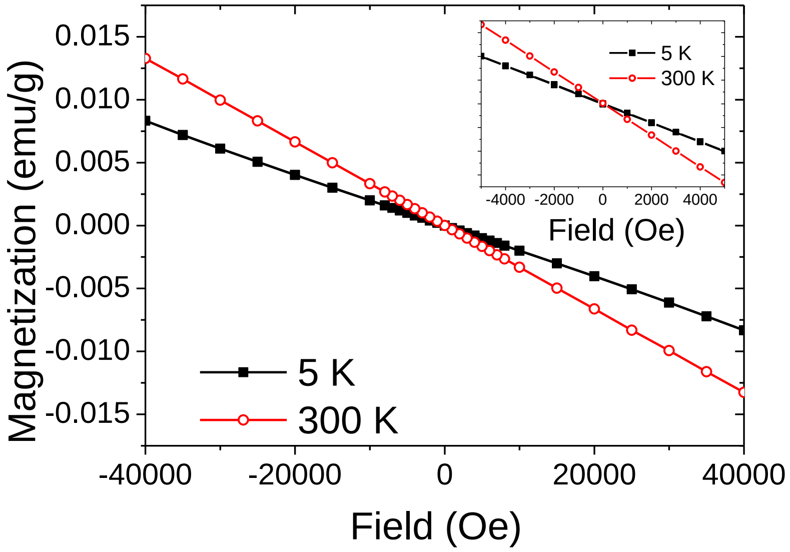

Figure S2: Field magnetization (open symbols) of pristine 6H-SiC measured at 5 K and 300 K. The sample is primarily diamagnetic. However, there is a difference in magnetic susceptibility at 5 K and

300 K. A slight deviation from the linear dependence of magnetization on field is observed at 5 K, which indicates a small paramagnetic contribution. The inset shows the zoom of the corresponding curves at the low field part. At both temperatures, the sample does not show ferromagnetic hysteresis.

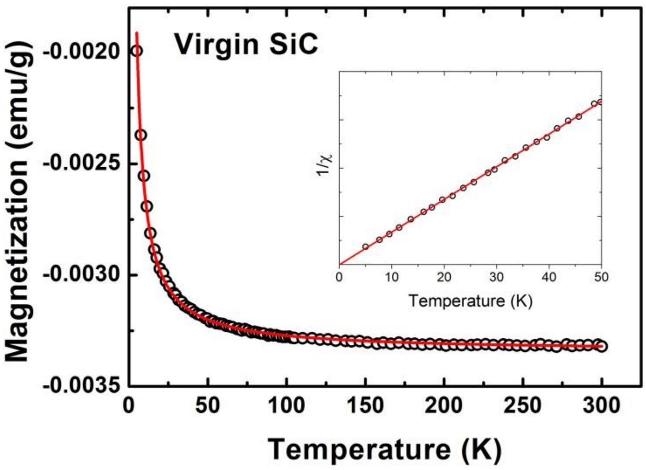

Figure S3: Temperature dependent magnetization (open symbols) of pristine 6H-SiC measured under a field of 10000 Oe. The result (open circles) can be fitted well (red solid line) by considering a paramagnetic contribution (Curie's law) with a diamagnetic background. The inset shows inverse susceptibility (after subtracting the diamagnetic background) *vs.* temperature with a linear, purely paramagnetic behavior with no sign of magnetic ordering.

In this part, we present the magnetic properties of pristine SiC. Figure S3 shows the field dependent magnetization (open symbols) of pristine 6H-SiC measured at 5 K and 300 K. The sample is primarily diamagnetic. However, there is a difference in magnetic susceptibility at 5 K and 300 K. A slight deviation from the linear dependence of magnetization on field is observed at 5 K, which indicates a small paramagnetic contribution. The inset shows the zoom of the corresponding curves at the low field part: there is no ferromagnetic hysteresis. The weak paramagnetism in pristine SiC is due to the intrinsic defects, which have been identified by electron spin resonance spectroscopy [2, 3]. In the following, we further confirm that the substrate we used for this study contains ONLY spin ½ paramagnetic center by magnetization

measurements, without any ferromagnetic inclusions as often observed in graphite [4].

Figure S4 shows the temperature dependent magnetization measured under a field of 10000 Oe. The large contribution represents the diamagnetic background, which is essentially temperature independent. We can well fit the curve by Curie's law:

$$M = M_0 + C\frac{B}{T} \quad (1)$$

Where M_0 is the diamagnetic background, B is the magnetic field, T is the temperature and C is the Curie constant. The inset shows the 1/M vs. temperature. The curve shows a linear behavior crossing the zero point, indicating the non-interacting nature between the paramagnetic centers.

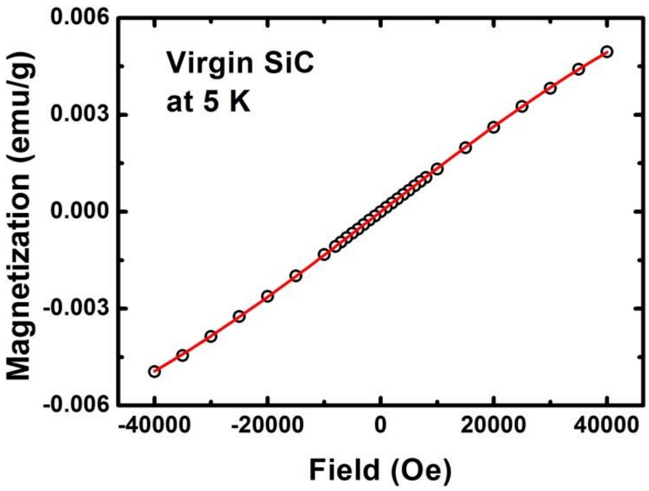

Figure S4: Fitting of the paramagnetism in the SiC substrate by Brillouin function with J=S=1/2.

The field-dependent magnetization of the pristine 6H-SiC was measured at 5 K. After subtracting the diamagnetic background, the result is shown in Fig. S5. No hysteresis loop is observed down to 5 K. The paramagnetism can be well fitted by a Brillouin function:

$$\mathrm{M} = NJ\mu_B g_J\left[\frac{2J+1}{2J}\coth\left(\frac{2J+1}{2J}\alpha\right) - \frac{1}{2J}\coth\left(\frac{\alpha}{2J}\right)\right] \quad (2),$$

where $\alpha = g_J J \mu_B H / k_B T$, the g_J factor is about 2 as obtained from electron spin resonance measurement [2, 3], μ_B is Bohr magneton, and N is the number of spins. The curve can only be well fitted by J = S = 0.5 corresponding to free electron spins. Thus in the pristine sample, the paramagnetic part is purely spin ½ paramagnetism, which is due to the intrinsic defects in SiC [2, 3].

Section 3: UV Raman results: no graphite or graphene formation

As ion irradiation induces damage only near the surface, UV-Raman spectroscopy with a He-Cd laser of 325 nm is used to characterize the structure after ion irradiation. Figure S2 exhibits UV-Raman spectra measured at room temperature for samples 5E12, 1E13, 5E13, 1E14 and the pristine sample, respectively. The corresponding phonon modes in 6H-SiC are identified [5] and no secondary phase is detected within the measurement sensitivity. The strength of the peaks decreases with increasing fluence, which indicates that the crystal structure is damaged upon ion bombardment. The samples 5E12 and 1E13 maintain relatively good crystallinity, while the structures of samples 5E13 and 1E14 suffer too much damage so that the sharp peaks vanish.

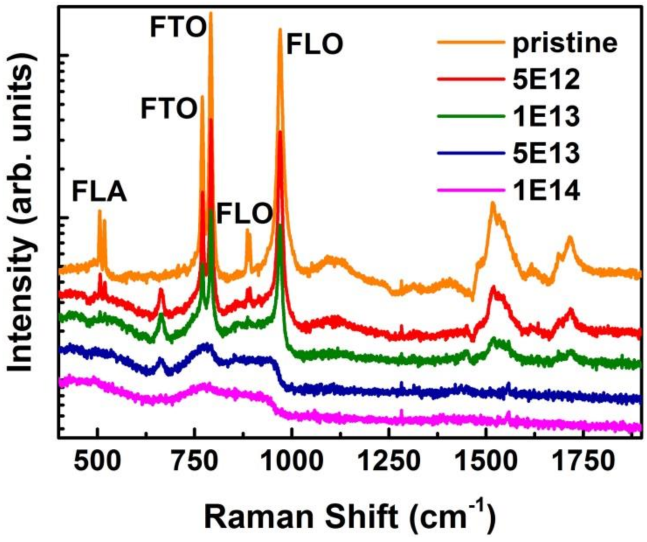

Figure S5: Room temperature UV-Raman spectra of samples 5E12, 1E13, 5E13, 1E14 as well as the pristine sample. The folded transverse acoustic (FTA) and optic (FTO) and longitudinal acoustic (FLA)

and optic (FLO) modes in 6H-SiC are identified in these spectra. The peaks at higher wavenumbers are second order modes.

Section 4: Divacancy identified in the ion implanted sample

In order to clarify the nature of defects which were created by the Xe ion implantation, positron annihilation Doppler broadening spectroscopy (DBS) was applied. DBS is an excellent technique to detect open volume defects from clusters consisting of several vacancies down to a mono-vacancy. The positron in a crystal lattice is strongly subjected to repulsion from the positive atom core. Because of the locally reduced atomic density inside the open volume defects, with a lower local electron density, positrons have a high probability to be trapped and to annihilate with electrons in these defects by the emission of two 511 keV photons. Monitoring of the 511 keV annihilation radiation was performed by DBS. The Doppler broadening of the 511 keV annihilation line is mainly caused by the momentum of the electron due to the very low momentum of the thermalized positron. There is one main parameters, S (shape), obtained from the 511 keV annihilation line. The S parameter reflects the fraction of positrons, annihilating with electrons of low momentum (valance electrons). Therefore, the S parameter is mainly a measure for the open volume in the material. The S parameter is defined as the ratio of the counts from the central part of the annihilation peak (here 510.17 keV – 511.83 keV) to the total number of counts in the whole peak (498 keV – 524 keV). DBS measurements were carried out with the mono-energetic slow positron beam ‘‘SPONSOR’’ at HZDR [6] at which a variation of the positron energy E from 30 eV to 36 keV with a smallest step width of 50 eV, if required, is possible. The energy resolution of the Ge detector at 511 keV was (1.09 $\pm$ 0.01) keV, resulting in a high sensitivity to changes in material properties from surface to depth of several µm.

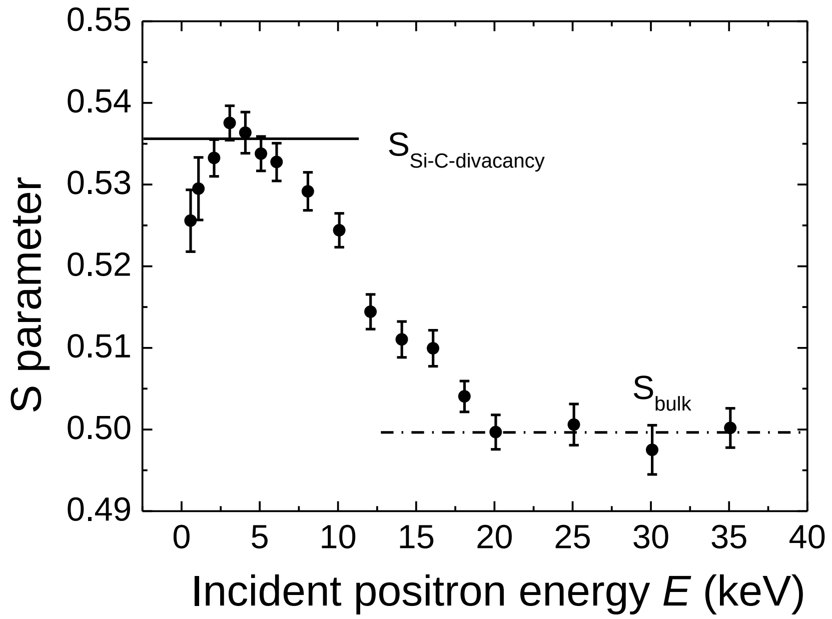

Figure S6: S parameter versus incident positron energy of the Xe implanted 6H-SiC sample.

Figure S6 shows the measured S parameter versus the incident positron energy. The maximum of the S parameter around positron energy of 3 – 4 keV corresponds to positron annihilation in the defects created by ion implantation within a depth of about 200 nm. For higher positron energies the positron annihilation is shifted more and more to the undamaged bulk material, the S parameter decreases and finally reaches the bulk value at 20 keV (dash-dot line in Fig. S6). The difference of the S values between the undamaged bulk S_{bulk} and the defects S_{defect} is a measure for type and concentration of these defects. Comprehensive investigations of defects in 6H-SiC were already done in the past. A relation between the S parameter and the number of agglomerated Si-C divacancies is published in [7] and shown as a scaling curve. From this scaling curve the S parameter of the Si-C divacancy was taken and plotted as a solid line in Fig. S6. It is clearly visible that the S parameter of the defects in the implanted range agrees well with the value of S for the Si-C divacancy which leads to the conclusion that Si-C divacancies were created by the Xe implantation into 6H-SiC.

Reference

1. P. Esquinazi, J. Barzola-Quiquia, D. Spemann, M. Rothermel, H. Ohldag, N. García, A. Setzer, and T. Butz, J. Magn. Magn. Mater. **322**, 1156 (2010).

2. N. T. Son, P. N. Hai, Mt. Wagner, W. M. Chen, A. Ellison, C. Hallin, B. Monemar, and E. Janzén, Semicond. Sci. Technol. **14**, 1141 (1999).

3. N. T. Son, B. Magnusson, Z. Zolnai, A. Ellison, and E. Janzén, Mat. Sci. Forum **437**, 457 (2004).

4. M. Venkatesan, P. Dunne, Y. H. Chen, H. Z. Zhang, and J. M. D. Coey, Carbon **56**, 279 (2013).

5. S. Nakashima and H. Harima, Phys. Status Solidi A **162**, 39 (1997).

6. W. Anwand, G. Brauer, M. Butterling, H.-R. Kissener and A. Wagner, Defect Diffus. Forum **331**, 25 (2012).

7. W. Anwand, G. Brauer, W. Skorupa, Appl. Surf. Sci. **194**, 131 (2002).
